# Assessing the Suitability of Habitats for *Porphyrio porphyrio indicus* and *Amaurornis phoenicurus* in Urban Wetlands of Peninsular Malaysia

**DOI:** 10.21315/tlsr2022.33.2.3

**Published:** 2022-07-15

**Authors:** Oluwatobi E. Olaniyi, Chukwuemeka O. Martins, Mohamed Zakaria

**Affiliations:** 1 Department of Forest Science and Biodiversity, Universiti Putra Malaysia, 43400 Serdang, Selangor, Malaysia; 2 Department of Ecotourism and Wildlife Management, Federal University of Technology, Akure, Nigeria

**Keywords:** Urban Wetlands, Climate Variability, Hydrology, Rails, Peninsular Malaysia, Tanah Paya Bandar, Perbezaan Iklim, Hidrologi, Rel, Semenanjung Malaysia

## Abstract

It becomes imperative to understand the eco-climatic predictors and know the suitable habitat for *Porphyrio porphyrio indicus* and *Amaurornis phoenicurus* in the urban wetlands to prevent their local extinction. The study explored the habitat suitability for *Porphyrio porphyrio indicus* and *Amaurornis phoenicurus* in Paya Indah wetlands and Putrajaya wetlands of Peninsular Malaysia. *Porphyrio porphyrio indicus* and *Amaurornis phoenicurus surveyed using the* point count technique, and a stratified random design. The maximum entropy modelling (MEM) approach and geographic information systems employed to determine the influence of 17 eco-climatic factors on the suitable habitats for the species. Water at a minimum depth (44.30%) and rainfall (74.20%) contributed to the availability of suitable habitats for *Porphyrio porphyrio indicus* in Paya Indah and Putrajaya wetlands. Also, dissolved oxygen (56.60%) and salinity (43.50%) contributed to habitat suitability for *Amaurornis phoenicurus* in Paya Indah and Putrajaya wetlands. Large portions of the two urban wetlands were unsuitable for the *Porphyrio porphyrio indicus* and *Amaurornis phoenicurus* populations because of several eco-climatic factors. Thus, the models as management tools with a robust population monitoring database and framework would enhance the management effectiveness of the two species and urban wetlands.

## Abstract (ms)

Pemahaman terhadap ramalan eko-iklim dan mengetahui habitat yang sesuai untuk *Porphyrio porphyrio indicus* dan *Amaurornis phoenicurus* di tanah paya bandar adalah penting untuk mengelakkan kepupusan. Kajian ini meneroka kesesuaian habitat untuk *Porphyrio porphyrio indicus* dan *Amaurornis phoenicurus* di tanah paya Paya Indah dan tanah paya Putrajaya di Semenanjung Malaysia. *Porphyrio porphyrio indicus* dan *Amaurornis phoenicurus* ditinjau menggunakan teknik kiraan titik dan reka bentuk rawak berstrata. Pendekatan pemodelan entropi maksimum (MEM) dan sistem maklumat geografi yang digunakan untuk menentukan pengaruh 17 faktor eko-iklim pada habitat yang sesuai untuk spesies tersebut. Air pada kedalaman minimum (44.30%) dan hujan (74.20%) menyumbang kepada ketersediaan habitat yang sesuai untuk *Porphyrio porphyrio indicus* di tanah paya Paya Indah dan tanah paya Putrajaya. Oksigen terlarut (56.60%) dan kadar kemasinan (43.50%) juga menyumbang kepada kesesuaian habitat untuk *Amaurornis phoenicurus* di tanah paya Paya Indah dan Putrajaya. Kebanyakan bahagian kedua-dua tanah paya bandar tidak sesuai untuk *Porphyrio porphyrio indicus* dan populasi *Amaurornis phoenicurus* disebabkan beberapa faktor eko-iklim. Oleh itu, model-model yang digunakan sebagai alat pengurusan dengan pangkalan data dan rangka kerja pemantauan penduduk yang mantap akan meningkatkan keberkesanan pengurusan kedua-dua spesies dan tanah paya bandar.

**Highlights**

- Based on the habitat suitability modelling for *Porphyrio porphyrio indicus* and *Amaurornis phoenicurus*, it was concluded that vast areas of Paya Indah and Putrajaya wetlands were unsuitable for the survival and sustenance of the waterbird species’ populations.
- The occurrence and distribution of the waterbird species in the two urban wetlands were impacted by the landscape, climate and hydrological factors.
- The Putrajaya wetland provided a better environment for *Porphyrio porphyrio indicus* and *Amaurornis phoenicurus* populations than the Paya Indah wetland.

## INTRODUCTION

Urban wetlands are fragile environments with high vulnerability to ecological change and environmental stressors caused by anthropogenic activities and climatic variability or change (Kansiime *et al*. 2007; Ehrenfeld 2008; Brinkmann *et al*. 2020). The urban wetlands are one of the most diverse ecosystems across the country because of the vegetation phytosociological characteristics, unpredictable rainfall patterns, and occurrences of contiguous, different adjoining landscapes (Rajpar & Zakaria 2014). Malaysia harboured fascinating, extensive natural and artificial urban wetland habitats with a wide variety of waterbirds, especially swamphens, waterhens, moorhens and watercocks (Zakaria *et al*. 2009; Martins *et al*. 2017; BirdLife International 2020). Nine species of family Rallidae with the International Union of Conservation and Nature (IUCN) conservation status of “least concern” occur in Malaysia (Wetlands International 2018).

Purple swamphen (*Porphyrio porphyrio indicus*) and white-breasted waterhen (*Amaurornis phoenicurus*) are species belonging to one of the most diverse families of waterbirds in Peninsular Malaysia referred to as the family Rallidae (Rails, Gallinules and Coots). Apart from Malaysia, *Porphyrio porphyrio indicus* distributed in South and Southeast Asia, Oceania, the Middle East, sub-Saharan Africa, Australia and the Mediterranean basin (Bara *et al*. 2014; Taylor 2016; Mundkur *et al*. 2017). The species associated with wetlands and dense marsh vegetation containing *Phragmites* spp. and *Typha* spp. (Taylor & Van Perlo 1998; Pearlstine & Ortiz 2009). *Amaurornis phoenicurus* connected to swampy environments and has a smaller range distributed across South and Southeast Asia (BirdLife International 2016).

The *Porphyrio porphyrio indicus* and *Amaurornis phoenicurus* do not approach the threshold for “vulnerable” under the range size criterion and classified as “least concern” (BirdLife International 2016). Habitat loss, invasive species and human interventions within the urban wetland ecosystems in Malaysia threatened the two species. The species distribution, vegetation dynamics, water and food resources, protection from predators and climatic conditions in the urban wetlands exacerbates the risk of local extinction (Zakaria & Rajpar 2010; Rajpar & Zakaria 2014). Even Wainger and Mazzotta (2011), Salari *et al*. (2014) and Gumbricht *et al*. (2017) opined that the limited knowledge on the wetlands’ environmental and climatic characteristics hindered a proper understanding of the distribution and management effectiveness of their biological resources.

Based on this premise, it has become imperative that suitable habitats with immense scientific value and eco-climatic predictors for the survival and sustenance of *Porphyrio porphyrio indicus* and *Amaurornis phoenicurus* populations in the urban wetlands of Malaysia determined. Habitat suitability model (HSM) development remains an effective tool for determining the ecological and microclimatic factors that influence waterbird species’ distribution within the wetlands in an urban setting. In the past three decades, the usage of HSMs to predict the likelihood of species occurrence (Hirzel & Lay 2008) or presence/absence (Rushton *et al*. 2004) or distribution (Franklin 1995; Guisan & Zimmermann 2000) has increased as it relates to environmental variables in a particular area. Several approaches involved in the variable selection of factors during habitat suitability modelling.

These approaches include climatic envelopes (Busby 1991; Carpenter *et al*. 1993; Hijmans *et al*. 2001), multivariate adaptive regression splines (Friedman 1991), Bayesian approach (Tucker *et al*. 1997), genetic algorithms (Stockwell & Peters 1999), random forest (Breiman 2001a; 2001b), artificial neural networks (Pearson *et al*. 2002; Thuiller 2003; Thuiller *et al*. 2009), generalised additive models (Thuiller 2003; Thuiller *et al*. 2009), generalised regression analysis and spatial prediction (Lehmann *et al*. 2003), generalised linear models (Thuiller 2003; Thuiller *et al*. 2009), classification, and regression trees (Thuiller 2003; Thuiller *et al*. 2009), ecological niche factor analysis (Hirzel *et al*. 2001; David & Stockwell 2006), maximum entropy (Phillips *et al*. 2006), generalised dissimilarity modelling (Ferrier *et al*. 2007), and support vector machines (Vapnik & Izmailov 2018).

Amidst these approaches, some employed presence only or presence and absence background data to predict species’ distribution. Hence, these approaches involved in various studies on habitat suitability modelling of waterbirds. For instance, Vallecillo *et al*. (2016) investigated the factors that influenced these avian groups’ distribution using the maximum entropy. Elith *et al*. (2006) and Aguirre-Gutiérrez *et al*. (2013) argued that the method offered better predictive performance, especially for presence-only data using background data and model performance evaluation. This method used the background data to account for sampling bias and to resolve non-detectability, false absences or false negatives associated with mobile organisms (Phillips *et al*. 2006), such as waterbird species.

Several authors employed the capability of maximum entropy to assess the habitat suitable for waterbirds across the globe. For instance, Kassara *et al*. (2017) predicted the suitability of wintering grounds for Eleonora’s falcons in Madagascar by integrating satellite and Global Positioning System data. Wang *et al*. (2019) determined the suitable habitat for conserving the red-crowned crane and migratory waterfowls in the Nenjiang River basin of Northeast China. Li *et al*. (2021) determined the wintering Anatidae habitats in the Gorges Reservoir Region, China. Nagy *et al*. (2021) explored maximum entropy with other three statistical modelling techniques (generalised linear model, boosted regression tree gradient boosted machine) to assess the climate change impact on waterbird species in the African-Eurasian flyways. Neice and McRae (2021) mapped the habitat suitable for the Eastern Black Rail throughout its Atlantic coastal range using the maximum entropy. Therefore, this research explored the maximum entropy to determine the eco-climatic predictors and habitats suitable for *Porphyrio porphyrio indicus* and *Amaurornis phoenicurus* in urban wetlands (Paya Indah and Putrajaya) of Peninsular Malaysia.

## MATERIALS AND METHODS

### Study Areas

**Figure 1:**
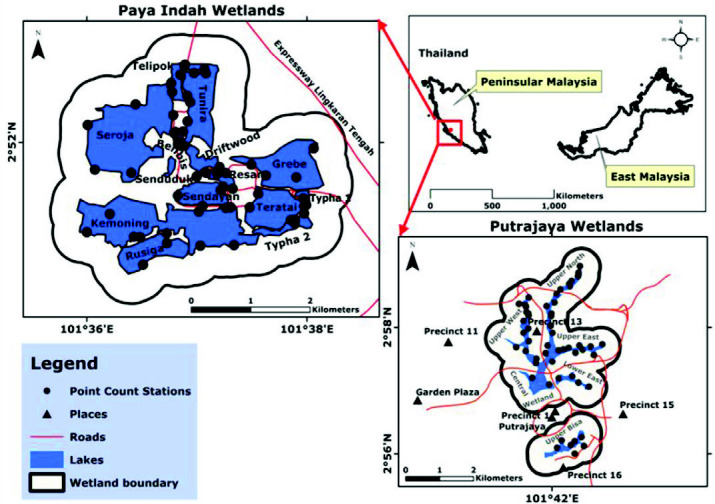
Paya Indah and Putrajaya wetlands of the Peninsular Malaysia.

The study undertook in the Paya Indah and Putrajaya wetlands of Peninsular Malaysia (Fig. 1). Paya Indah and Putrajaya wetlands are the largest urban wetlands in the most developed state (Selangor) and fastest-growing region (Putrajaya) of Peninsular Malaysia. Paya Indah wetland is located within 101°36.39′E to 101°36.85′E longitude and 2°51.35′N to 2°51.59N latitude, next to the administrative area of Putrajaya (Rajpar *et al*. 2017). It covers a landmass of 450 ha managed by the Department of Wildlife and National Park, Peninsular Malaysia (Salari *et al*. 2014). It comprises of 14 ex-tin mine water ponds, a disturbed forest and an undisturbed peat swamp forest (Rajpar & Zakaria 2012). It has five land use/land cover (LULC) classes – marsh swamps, a lotus swamp, a lake, an open area with scattered trees, and scrublands (Rajpar *et al*. 2017), and 20 waterbird species recorded in the wetland (Zakaria & Rajpar 2010).

Putrajaya wetland is located within 101°41.90′E to 101°42.43′E longitude and 2°57.71′N and 2°57.81′N in Putrajaya of Peninsular Malaysia (Rajpar & Zakaria 2013). It covers a landmass of 200 ha with five LULC areas containing the planted area, open water, islands, inundated area and walking trails. The wetland comprises of 24 cells that primarily control the water level and trap pollutants derived from upstream sources from flowing into the catchment areas of the Chua and Bisa rivers. It comprises four vegetation classes of aquatic plants with emergent plants, fruiting trees, flowering trees and bushes, and shrubs (Rajpar & Zakaria 2013).

### Occurrence Data of the Species

The point count technique employed to collect the occurrence data of the two studied species – *Porphyrio porphyrio indicus* and *Amaurornis phoenicurus* (Bibby *et al*. 2000; Wretenberg *et al*. 2006; Tozer *et al*. 2010; Lloyd & Doyle 2011). The bird survey spanned from November 2016 to January 2019. Fifty-seven and 54 count stations in Paya Indah and Putrajaya wetlands placed based on their visibility, using binoculars with at least 100 m intervals apart to avoid the double count of the same avian species in the same count station. Also, the double count avoided by involving two survey teams simultaneously within the same period at adjacent count stations. The bird count surveys in each count station with a maximum variable radius of 100 m for 10 min conducted from 0730 am to 1100 am. Hutto and Young (2002) recommended the ten-minute counts to reduce the number of birds ignored.

These surveys carried out four times within a week during the 26 consecutive months. The bird survey in each point count station done 20 times and pooled for analysis. The abundance dataset of each studied waterbird species pooled to understand its trend and avoid its random fluctuation. Because of the inability to reach the actual locations of the *Porphyrio porphyrio indicus* and *Amaurornis phoenicurus*, the distance between the observer and waterbird species measured at each count station using the Hypsometer (TruePulse R 200x model). The occurrence sites estimated based on the direction and distance between the observer and the studied waterbird species. The efficient design ensured a reduced bias with improved data accuracy and precision (Dunn *et al*. 2006). The data recorded in the survey are the name of the lake, species observed on the lake, the total number sighted, vegetation type, land use and time sighted.

### Data Acquisition and Modelling

**Figure 2:**
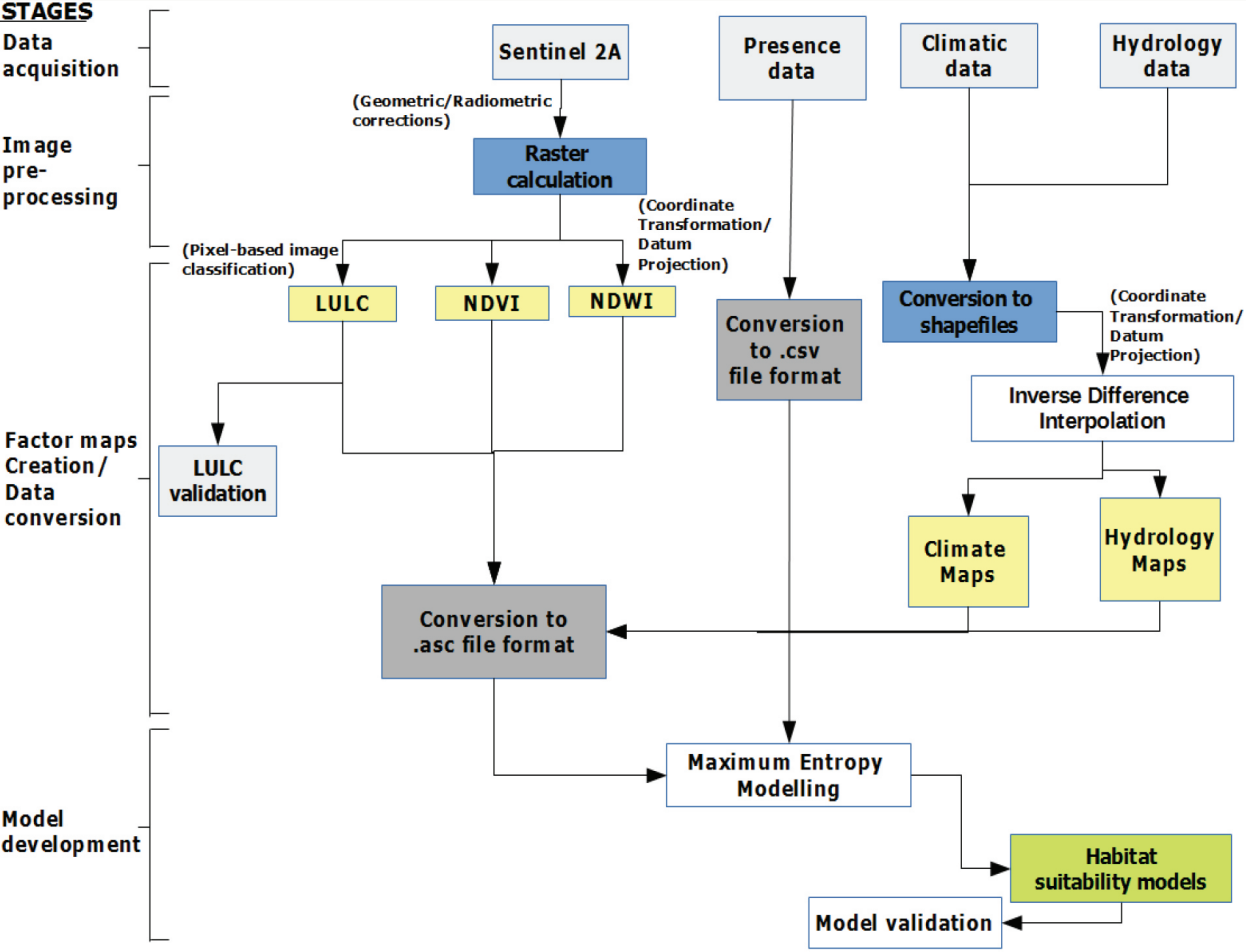
Framework for habitat suitability modelling of *Porphyrio porphyrio indicus* and *Amaurornis phoenicurus* in Paya Indah and Putrajaya wetlands of Peninsular Malaysia.

This study involved four stages of the modelling process (Fig. 2).

#### Data acquisition

Satellite imageries and climatic/hydrology data downloaded and collected to provide the primary source of data required as explanatory variables. The collection of ground truth points required for model validation. Sentinel 2A MSI Level-1C satellite imageries sourced from the United States Geological Survey (USGS) archives at scales of 10 m resolutions via Global Visualisation Viewer. These images captured during the driest month (9 April 2018) to minimise the interference from cloud cover and to depict the state of LULC and water coverage of the sites. The satellite data used as the principal source of data for the extraction of LULC, Normalized Difference Vegetation Index (NDVI) and Normalised Difference Water Index (NDWI).

Climatic factors (relative humidity, rainfall, wind speed, atmospheric pressure and atmospheric temperature) got from the three recording stations of the National Climate Center, Malaysian Meteorological Department, Malaysia. These recording stations are Kuala Lumpur International Airport, Sepang; Petaling Jaya; and Pusat Pert., Serdang. The distance between the recording stations and the study sites varied from 4.30 km to 27.73 km. The hydrological data (electrical conductivity, dissolved oxygen, water quality index) (House & Ellis 1987; Breaban *et al*. 2012), turbidity, temperature, salinity, pH, minimum depth and maximum depth) collected from the National Hydraulic Research Institute of Malaysia.

Three factors (water quality index, minimum depth and maximum depth) not included in the habitat suitable modelling for the two studied species in Putrajaya wetlands. This limitation caused by non-available data at the National Hydraulic Research Institute of Malaysia and the National Climate Center, Malaysian Meteorological Department, Malaysia. The hydrological and climatic data collected from November 2016 to January 2019 to coincide with the study period. However, the seventeen eco-climatic factors (Table 1) are LULC, NDVI, NDWI, electrical conductivity, dissolved oxygen, water quality index, turbidity, temperature, salinity, pH, minimum depth, maximum depth, relative humidity, rainfall, wind speed, atmospheric pressure and atmospheric temperature.

#### Image Pre-processing

The Sentinel 2A bands had already been atmospherically corrected with the bands in the dataset containing true top of atmosphere reflectance integer units. The raster subjected to geometric and radiometric corrections using histogram equalisation, haze and noise reduction functions in ERDAS Imaging 2014 software (ERDAS 2014). The spatial reference systems (World Geodetic System 1984) of the wetlands’ satellite imagery datasets and vector data (places, roads, lakes, boundaries) transformed and projected to the Malaysian local projected coordinate system (Selangor GDM 2000).

#### Creation of Factor Maps/Data Conversion

This study used four criteria (hydrology, climatic, waterscape, landscape) and seventeen factors to model the influence of eco-climatic factors on the spatial heterogeneity of waterbirds in Peninsular Malaysia. The two factors for landscape (LULC and NDVI; a measure of vegetation cover, forage availability and human activity) and one factor for waterscape (NDWI; a measure of water availability) selected, as suitability factors based on the studies by Hirzel *et al*. (2001); Brotons *et al*. (2004); Soulliere *et al*. (2007); Tian *et al*. (2008) and Tang *et al*. (2011). The NDVI and NDWI extracted from the Sentinel 2A imagery. Also, the pixel-based image classification using the supervised classification method employed to determine the LULC (a measure of safe shelter and forage availability for waterbirds) on the wetlands.

The United States Geological Survey (USGS) Land Classification Scheme (Anderson *et al*. 1976) changed into five and four LULC classes for the analysis based on the present LULC scenario in Paya Indah and Putrajaya wetlands. Paya Indah wetlands had a semi-closed secondary forest, shrub lands, marsh swamp/Lotus swamp/grassy vegetation, lakes and bare grounds/built-up areas. The Putrajaya wetland had a semi-closed secondary forest/aquatic herbaceous vegetation, marsh swamp/aquatic grassy vegetation, lakes, bare ground/built-up areas. A field survey carried out to gather ground-truthing to allow for accurate assessment. Hand-held GPS (GPS Map 78s, GARMIN) used to collect coordinates of points representing the different LULC classes. Ground truthing performed in April 2018 to collect a total of 190 ground control points in the study area during the season, similar to the acquisition of satellite datasets. The ground control points used as training samples during the LULC classification of the wetlands.

Error matrices and kappa statistics computed using the accuracy assessment tool in ERDAS Imagine 2014 software (ERDAS 2014). The presence and hydrology/hydrological data converted to delimit text and .asc file formats. The factor files (continuous and raster data) of the hydrological and climatic parameters created using the inverse difference interpolation method according to the procedure of Knight *et al*. (2005).

#### Construction of Habitat Suitability Model (Model Development and Validation)

The habitat suitability models (HSM) of the two waterbird species and their eco-climatic predictors were constructed and determined using the maximum entropy modelling approach (Phillips *et al*. 2006). The presence/absence data (number of individual waterbirds detected and non-detected, 75%) and the 17 eco-climatic factors served as the dependent and independent variables. Twenty-five per cent of the presence data (abundance of the waterbirds) and the 1000 background or pseudo-absence points were employed to validate the models (Phillips *et al*. 2006). For this study, a minimum contribution of 10% set as the level for an ecologically meaningful contribution of the eco-climatic factors to habitat suitability of *Porphyrio porphyrio indicus* and *Amaurornis phoenicurus*.

Each of the generated HSM (a continuous raster file) reclassified into five suitability classes from highly non-suitable (1) to highly suitable (5) based on the waterbird habitat suitability continuum framework developed by Dong *et al*. (2013) using Jenk’s natural breaks (Jenk 1967). The habitat suitability map for each species categorised into five suitability classes (based on Jenk’s Natural Breaks Classification): Highly suitable, suitable, moderately suitable, non-suitable and highly non-suitable. According to Hosmer and Lemeshow (2000), the area under the curve value within the range of 0.50 to 0.70, 0.70 to 0.90, and greater than 0.90 showed that the model accuracy is low, moderate and high. The area under the curve value of > 0.75 is considered more accurate, acceptable, and suitable for predicting species distribution (Elith 2000; Vanagas 2004; Phillips *et al*. 2006; Lobo *et al*. 2008).

## RESULTS

**Figure 3:**
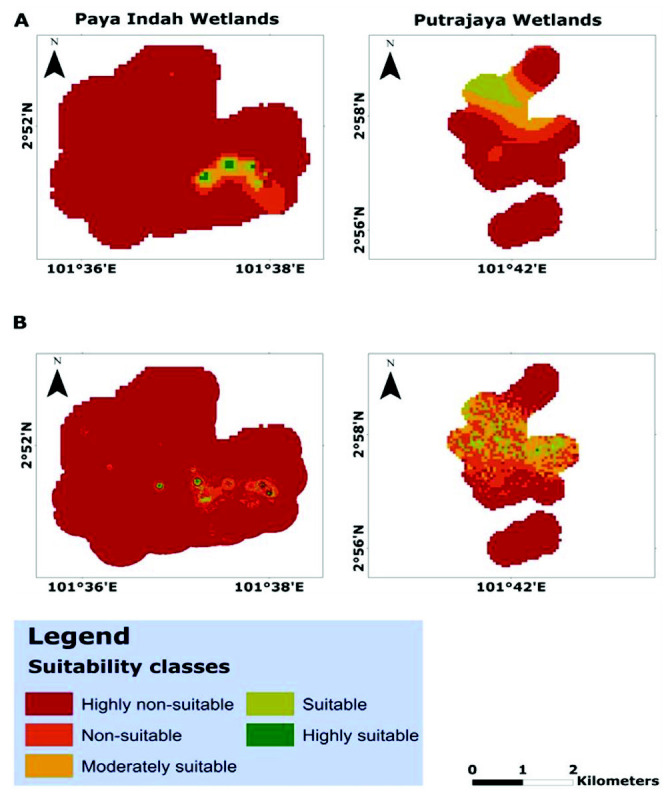
Habitat suitability models of (A) *Porphyrio porphyrio indicus* and (B) *Amaurornis phoenicurus* in Paya Indah and Putrajaya wetlands of Peninsular Malaysia.

Fig. 3 presented the fitted habitat suitability models for studied species in Paya Indah and Putrajaya wetlands. The four models had a robust performance, with the area under the curve values greater than 0.50 of a random model. The area under the curve values for *Porphyrio porphyrio indicus* in Paya Indah and Putrajaya wetlands were 0.987 and 0.939. Also, the area under the curve values for *Amaurornis phoenicurus* in Paya Indah and Putrajaya wetlands were 0.896 and 0.993.

Table 1 shows the eco-climatic predictors of habitat suitable for *Porphyrio porphyrio indicus* and *Amaurornis phoenicurus* in Paya Indah and Putrajaya wetlands. The range of electric conductivity (15.49uS/cm–41.18uS/cm), dissolve oxygen (4.47 mg/L–8.22 mg/L), turbidity (2.02 NTU–23.73 NTU), water temperature (24.45°C–30.79°C), salinity (0.50 ppt–5.04 ppt), minimum water depth (0.65 m–5.92 m), maximum depth (3.12 m–20.74 m), relative humidity (27.855%–77.530%), rainfall (9.976 mm–10.691 mm), wind speed (1.487 m/s–1.618 m/s) and atmospheric temperature (27.741°C–27.773°C) offered suitable habitats for the two species (Table 1).

Table 2 presents the eco-climatic factors’ contribution to the habitat suitability modelling of *Porphyrio porphyrio indicus* and *Amaurornis phoenicurus* in Paya Indah and Putrajaya wetlands. Based on the maximum entropy modelling result, minimum water depth (m) had the highest contribution (44.30%) to the habitat suitable for *Porphyrio porphyrio indicus* in Paya Indah wetland, followed by dissolved oxygen (24.90%). In Putrajaya wetland, rainfall (mm) had the highest contribution (74.20%) to the habitat suitable for *Porphyrio porphyrio indicus* in Paya Indah wetland, followed by pH (11.10%). The landscape and waterscape factors did not influence the habitat suitability for *Porphyrio porphyrio indicus* in the Putrajaya wetland. However, three and two eco-climatic factors predicted the habitat suitability for *Porphyrio porphyrio indicus* in Paya Indah and Putrajaya wetlands.

As regards *Amaurornis phoenicurus*, dissolved oxygen (mg/L) had the highest contribution (56.60%) to its habitat suitability in Paya Indah wetland, followed by water quality index (31.70%). At Putrajaya wetland, salinity (ppt) had the highest contribution (43.50%) to the habitat suitable for *Amaurornis phoenicurus*, followed by LULC (27.90%). Thus, three eco-climatic factors predicted the habitat suitability for *Amaurornis phoenicurus* in Paya Indah and Putrajaya wetlands. A few eco-climatic factors had no significant influence on the suitable habitat for *Porphyrio porphyrio indicus* and *Amaurornis phoenicurus* in Paya Indah and Putrajaya wetlands. The eco-climatic factors are: the waterscape (NDWI), landscape (NDVI), climatic (relative humidity, wind speed, atmospheric pressure, atmospheric temperature) and hydrological (electric conductivity, turbidity, water temperature).

Fig. 3 and Table 3 present the habitat suitability models and their attributes for *Porphyrio porphyrio indicus* and *Amaurornis phoenicurus* in Paya Indah and Putrajaya wetlands. From the habitat suitability map for *Porphyrio porphyrio indicus*, the highly non-suitable area occupied the highest wetland area (1459.56 ha; 92.40%) in the Paya Indah wetland. The highly suitable area occupied the lower wetland area (5.61 ha; 0.36%). For *Porphyrio porphyrio indicus* in Putrajaya wetland, the highly non-suitable area occupied the highest wetland area (959.24 ha; 67.54%), while there was no wetland coverage for the highly suitable area. The highly non-suitable area for *Amaurornis phoenicurus* in Paya Indah and Putrajaya wetlands occupied the highest wetland area (1507.54 ha; 95.44%) and (701.46 ha; 49.39%). The highly non-suitable area by *Amaurornis phoenicurus* occupied the lower wetland area (<1%) in the two urban wetlands.

## DISCUSSION

Since the 20th century began, the decline in the range and population size of waterbird species attributed to the loss of suitable habitat and excessive human activities such as hunting, agriculture and urbanisation (BirdLife International 2004). BirdLife BHDTF (2013) opined that the sufficient and large suitable areas for birds are essential for sustaining a healthy population in the long term. In this study, large portions of the two urban wetlands are unsuitable for the survival and sustenance of *Porphyrio porphyrio indicus* and *Amaurornis phoenicurus* populations. The small suitable habitat for the two species in Paya Indah and Putrajaya wetlands calls for urgent attention despite the initial scientific notion of their wide range in Malaysia and conservation status of “least concern” to prevent their local extinction. The ambient ecological and climatic factors attributed to this less suitable habitat. This assertion corroborated the findings of Esper *et al*. (2012), Ismail and Rahman (2013) and Jordan (2017) that habitat characteristics and climatic factors affected the distribution, breeding activities and suitable habitat for waterbirds.

There is inadequate information on the influence of microclimatic and ecological variables on habitat suitability for *Porphyrio porphyrio indicus* and *Amaurornis phoenicurus* in any urban wetland. However, this study revealed the occurrence and distribution of *Porphyrio porphyrio indicus* and *Amaurornis phoenicurus* in Paya Indah and Putrajaya wetlands, influenced by a few landscapes, climatic and hydrological factors. In contrast, waterscape (NDWI; a measure of water availability) had no significant influence on the two species distribution in both wetlands. For the first species (*Porphyrio porphyrio indicus*), the landscape of Putrajaya wetland contained a more suitable habitat for its populations than the Paya Indah wetland due to varying eco-climatic predictors. Three hydrological factors (minimum depth, dissolved oxygen and maximum depth) contributed to *Porphyrio porphyrio indicus* distribution in the Paya Indah wetland.

The climatic (rainfall) and hydrological factors (water pH) contributed to *Porphyrio porphyrio indicus* distribution in the Putrajaya wetland. Paya Indah and Putrajaya wetlands have experienced one form of human activities or the other over the years. Zakaria and Rajpar (2010) and Hassen-Aboushiba (2015) reported that tin mining and agricultural activities, coupled with tourism infrastructural development, were the major anthropogenic activities in the Paya Indah wetland. Also, urban sprawl and water purification/supply are among the drivers of landscape dynamics in Putrajaya Wetland. The anthropogenic activities within the two urban wetlands associated with the varied eco-climatic predictors related to habitat suitability for *Porphyrio porphyrio indicus*. This view supported Bai *et al*. (2013) and Li *et al*. (2018) that the wetlands converted to other land use types could influence their microclimates and alter their hydrological cycle.

In Paya Indah wetland, the minimum and maximum depths of the lakes contributed to making Paya wetland a less suitable habitat for the *Porphyrio porphyrio indicus* populations, as this affects the wetlands’ habitat characteristics, forage availability and bird locomotion. It aligned with the views of Rajpar and Zakaria (2011) that water depth influenced the occurrence, diversity, and distribution of waterbirds in the Paya Indah wetland. Fluctuation in water level might alter habitat characteristics and cause changes in waterbirds communities (Lee *et al*. 2006; Johnson *et al*. 2007). Besides the limiting access to foraging habitats, water depth also affects the net energy intake of *Porphyrio porphyrio indicus* because foraging efficiency decreases with increasing water depth. Gawlik (2002) showed that the locomotion of wading birds foraging prey in the water column slowed down in deep water as water resistance increases with depth.

Rainfall was the only climatic variable that influenced the habitat suitable for *Porphyrio porphyrio indicus* in the Putrajaya wetland. The lower amount of rainfall in Putrajaya wetland than Paya Indah wetland contributed to the suitability of the habitat for the survival and sustenance of *Porphyrio porphyrio indicus* populations. This assertion supports the findings of Wormworth and Mallon (2014) that rainfall is a limiting factor to the birds’ distribution because of their direct impact on wetland habitats. Water pH is also a contributory factor to the suitable habitat for *Porphyrio porphyrio indicus* in the Putrajaya wetland. Water pH (ranging between 7.2 and 9.2) in Putrajaya wetland was alkaline but tended towards the neutral level during the entire study period. The neutral waters in the Putrajaya wetland connected to its more suitable habitat for *Porphyrio porphyrio indicus* than the Paya Indah wetland. Longcore *et al*. (2006) reported that a water pH in the alkaline range supported higher macro-invertebrates and attracted more waterbirds to the water bodies. Also, Minns (1989) considered pH an indicator of overall productivity that could cause habitat diversity with a significant influence on the species richness of phytoplankton (food for *Porphyrio porphyrio indicus*).

Water salinity contributed to a more suitable habitat for *Amaurornis phoenicurus* in the Putrajaya wetland than in the Paya Indah wetland. The direct relationship between water salinity and food resources in urban wetlands associated with water salinity. According to Ma *et al*. (2010), water salinity determined the spatial heterogeneity of aquatic animals and zoobenthos and influenced the locality of foraging sites by waterbird species. But, the landscape (LULC; a measure of vegetation cover, forage availability and human activity) had a significant influence on the suitable habitat for *Amaurornis phoenicurus* in the two urban wetlands.

It depicted the roles of anthropogenic activities (the driver of LULC), forage availability, and vegetation cover in the occurrence and distribution of *Amaurornis phoenicurus*. Van Niekerk (2010), Mundava *et al*. (2012), Tanalgo *et al*. (2015), Catford *et al*. (2017) and Marasinghe *et al*. (2020) opined that anthropogenic pressure poses a threat to the population growth and habitat suitability of waterbirds. Hence, our findings support the findings made by Rajpar and Zakaria (2014) and Jahanbakhsh *et al*. (2017) that vegetation covers affected habitat selection, distribution and diversity of waterbirds in Putrajaya wetland, Malaysia and Parishan International wetland, Iran.

The dissolved oxygen was the only eco-climatic (hydrological) factor that influenced the habitat suitability of both species (*Porphyrio porphyrio indicus* and *Amaurornis phoenicurus*) in the Paya Indah wetland. For this reason, the dissolved oxygen link to *Porphyrio porphyrio indicus* and *Amaurornis phoenicurus* distribution is non negligible. According to Sathe *et al*. (2001), dissolved oxygen is vital in regulating the metabolic processes of aquatic plants. It is an indicator of ecosystem health in a wetland ecosystem. Previous studies observed a significant relationship between dissolved oxygen and waterbirds (Thapa & Saund 2012; Sulai *et al*. 2015; Haq *et al*. 2018).

Thus, the influence of eco-climatic variability on the urban wetlands’ vegetation composition, structure, hydro-morphological properties, and consequently the populations’ distribution and sustainability of *Porphyrio porphyrio indicus* and *Amaurornis phoenicurus* is significant. According to Sekercioglu *et al*. (2012), Porte and Gupta (2017), and Mundkur *et al*. (2017), this global phenomenon had broad impacts on the distribution, morphology, carrying capacities and seasonal variations of urban wetlands connected to the feeding and breeding activities of waterbird species such as *Porphyrio porphyrio indicus* and *Amaurornis phoenicurus*.

## CONCLUSIONS

Our findings revealed that large portions of the two urban wetlands were not suitable for the survival and sustenance of *Porphyrio porphyrio indicus* and *Amaurornis phoenicurus* populations. Notwithstanding, the Putrajaya wetland offered a more suitable habitat to the *Porphyrio porphyrio indicus* and *Amaurornis phoenicurus* populations as to the Paya Indah wetland. The habitat suitability associated with the favourable rainfall, water pH, salinity, and LULC of Putrajaya wetland to *Porphyrio porphyrio indicus*. *Porphyrio porphyrio indicus* thrived more in urban wetlands with lower rainfall, neutral pH, higher dissolved oxygen, shallow water depth, and marsh swamp/lotus swamp/grassy vegetation interspersed with semi-closed secondary forest.

*Amaurornis phoenicurus* experienced high survival and suitable habitat with lower rainfall, higher dissolved oxygen, higher water quality index, lower salinity, and marsh swamp/lotus swamp/grassy vegetation interspersed with semi-closed secondary forest in urban wetlands. Landscape, climatic and hydrological factors influenced the occurrence and distribution of *Porphyrio porphyrio indicus* and *Amaurornis phoenicurus* in Paya Indah and Putrajaya wetlands. Thus, the developed habitat suitability models gave a better understanding of the current extent to which eco-climatic factors contributed to the habitat suitable for *Porphyrio porphyrio indicus* and *Amaurornis phoenicurus* in Paya Indah and Putrajaya wetlands of Peninsular Malaysia. The models as management tool adopted with a robust population monitoring database and framework will enhance the management effectiveness of the two species and urban wetlands.

## Figures and Tables

**Table 1:** Attributes of the environmental factors in Paya Indah and Putrajaya wetlands.

Parameters	Wetlands	

	Paya Indah	Putrajaya
Climatic		
Atmospheric pressure (Hpa)	1009.203–1009.325	1009.436–1009.935
Wind Speed (m/s)	1.487–1.618	1.361–1.383
Rainfall (mm)	9.976–10.691	8.525–9.027
Relative Humidity (%)	27.855–77.530	76.958–78.016
Atmospheric temperature (°C)	27.741–27.773	27.309–27.564
Hydrological		
Water temperature (°C)	24.45–30.79	29.94–30.72
pH	5.73–9.05	7.35–7.58
Dissolved oxygen (mg/L)	4.47–8.22	6.12–7.35
Electrical conductivity (uS/cm)	15.49–41.18	59.78–152.31
Salinity (ppt)	0.50–5.04	0.03–0.08
Turbidity (NTU)	2.02–23.73	12.67–76.85
Maximum depth(m)	3.12–20.74	Not Determined
Minimum depth (m)	0.65–5.92	Not Determined
Water quality index	50.66–80.24	Not Determined
Land use/land cover classes (LULC)		
Marsh swamp/lotus swamp/grassy vegetation	310.24 (19.64)	345.38 (24.31)
Semi-closed secondary forest	391.77 (24.80)	395.79 (27.87)
Shrubland	372.75 (23.60)	0.00 (0.00)
Bare ground/built-up areas	131.54 (8.33)	367.61 (25.88)
Lakes	373.25 (23.63)	311.57 (21.94)
Normalised difference water index (NDWI)		
Water areas	1175.23 (74.40)	1255.79 (88.41)
Non-water areas	404.32 (25.60)	164.56 (11.59)
Normalised difference vegetation index (NDVI)		
Vegetated areas	1159.96 (73.44)	1139.86 (80.25)
Non-vegetated areas	419.59 (26.56)	280.49 (19.75)

*Note*: Each cell in LULC, NDWI and NDVI signifies “land cover area in hectares” (proportion in %).

**Table 2:** The contribution of eco-climatic to the habitat suitability modelling of *Porphyrio porphyrio indicus* and *Amaurornis phoenicurus* in Paya Indah and Putrajaya wetlands.

Criteria	Factors	*Porphyrio porphyrio indicus*				*Amaurornis phoenicurus*			

		Paya Indah		Putrajaya		Paya Indah		Putrajaya	

		Factor contribution (%)	Rank	Factor contribution (%)	Rank	Factor contribution (%)	Rank	Factor contribution (%)	Rank
Hydrology									
	Electric conductivity (uS/cm)	0.00^ns^	9	1.40^ns^	5	0.00^ns^	4	0.00^ns^	7
	Dissolved oxygen (mg/L)	24.90*	2	4.90^ns^	4	56.60*	1	0.50^ns^	6
	Water quality index	4.10^ns^	4	NA	NA	31.70*	2	NA	NA
	Turbidity (NTU)	0.00^ns^	9	0.00^ns^	6	0.00^ns^	4	6.00^ns^	4
	Water temperature (°C)	0.00^ns^	9	0.00^ns^	6	0.00^ns^	4	2.40^ns^	5
	Salinity (ppt)	0.60^ns^	7	8.40^ns^	3	0.00^ns^	4	43.50*	1
	pH	0.70^ns^	6	11.10*	2	0.00^ns^	4	0.00^ns^	7
	Minimum Depth (m)	44.30*	1	NA	NA	0.00^ns^	4	NA	NA
	Maximum Depth (m)	23.30*	3	NA	NA	0.00^ns^	4	NA	NA
Climatic									
	Relative humidity (%)	2.00^ns^	5	0.00^ns^	6	0.00^ns^	4	0.00^ns^	7
	Rainfall (mm)	0.00^ns^	9	74.20*	1	0.00^ns^	4	19.60*	3
	Wind speed (m/s)	0.00^ns^	9	0.00^ns^	6	0.00^ns^	4	0.00^ns^	7
	Atmospheric pressure (Hpa)	0.00^ns^	9	0.00^ns^	6	0.00^ns^	4	0.00^ns^	7
	Atmospheric temperature (°C)	0.00^ns^	9	0.00^ns^	6	0.00^ns^	4	0.00^ns^	7
Waterscape									
	Normalised Difference Water Index	0.00^ns^	9	0.00^ns^	6	0.00^ns^	4	0.00^ns^	7
Landscape									
	Normalised Difference Vegetation Index	0.00^ns^	9	0.00^ns^	6	0.00^ns^	4	0.00^ns^	7
	Land use/land cover	0.10^ns^	8	0.00^ns^	6	11.70*	3	27.90*	2

*Notes*:

* signifies the parameter is significant (Factor contribution ≥ 10%) while ns signifies the parameter is non-significant (Factor contribution < 10%); “NA” means “Not Assessed”.

**Table 3:** Attributes of habitat suitability models for PPI and AP in Paya Indah and Putrajaya wetlands.

Suitability classes	*Porphyrio porphyrio indicus*				*Amaurornis phoenicurus*			

	Paya Indah		Putrajaya		Paya Indah		Putrajaya	

	Area (Ha)	Proportion (%)	Area (Ha)	Proportion (%)	Area (Ha)	Proportion (%)	Area (Ha)	Proportion (%)
Highly suitable	5.61	0.36	0.00	0.00	2.83	0.18	0.73	0.05
Suitable	17.54	1.11	102.39	7.21	11.20	0.71	126.35	8.90
Moderately suitable	28.07	1.78	162.66	11.45	16.90	1.07	241.08	16.97
Non-suitable	68.77	4.35	196.06	13.80	41.08	2.60	350.73	24.69
Highly non-suitable	1459.56	92.40	959.24	67.54	1507.54	95.44	701.46	49.39
Total	1579.55	100.00	1420.35	100.00	1579.55	100.00	1420.35	100.00
Specific locations/lakes with the suitable habitat	Southwest Grebe, Northern Teratai, Eastern Sendayan, Southeastern Kemoning		Upper West, Upper East, Southern Upper North		Teratai, Southwest Grebe, Western/Eastern Sendayan, Southeastern Kemoning		Upper West, Upper East, Lower East, Central Wetland	
